# Mouse Models and Techniques for the Isolation of the Diabetic Endothelium

**DOI:** 10.1155/2013/165397

**Published:** 2013-06-11

**Authors:** April L. Darrow, J. Gregory Maresh, Ralph V. Shohet

**Affiliations:** ^1^Center for Cardiovascular Research, University of Hawaii John A. Burns School of Medicine, Honolulu, HI 96813, USA; ^2^Department of Cell and Molecular Biology, University of Hawaii John A. Burns School of Medicine, Honolulu, HI 96813, USA; ^3^Department of Medicine, University of Hawaii John A. Burns School of Medicine, 651 Ilalo Street, Honolulu, HI 96813, USA

## Abstract

Understanding the molecular mechanisms underlying diabetic endothelial dysfunction is necessary in order to improve the cardiovascular health of diabetic patients. Previously, we described an *in vivo*, murine model of insulin resistance induced by feeding a high-fat diet (HFD) whereby the endothelium may be isolated by fluorescence-activated cell sorting (FACS) based on Tie2-GFP expression and cell-surface staining. Here, we apply this model to two new strains of mice, ScN/Tie2-GFP and *ApoE*(−/−)/Tie2-GFP, and describe their metabolic responses and endothelial isolation. ScN/Tie2-GFP mice, which lack a functional toll-like receptor 4 (TLR4), display lower fasting glucose and insulin levels and improved glucose tolerance compared to Tie2-GFP mice, suggesting that TLR4 deficiency decreases susceptibility to the development of insulin resistance. *ApoE*(−/−)/Tie2-GFP mice display elevated glucose and cholesterol levels versus Tie2-GFP mice. Endothelial isolation by FACS achieves a pure population of endothelial cells that retain GFP fluorescence and endothelial functions. Transcriptional analysis of the aortic and muscle endothelium isolated from *ApoE*(−/−)/Tie2-GFP mice reveals a reduced endothelial response to HFD compared to Tie2-GFP mice, perhaps resulting from preexisting endothelial dysfunction in the hypercholesterolemic state. These mouse models and endothelial isolation techniques are valuable for assessing diabetic endothelial dysfunction and vascular responses *in vivo*.

## 1. Introduction

Vascular complications are the main causes of morbidity and mortality associated with diabetes. Diabetics have a 2–4-fold increased risk for developing cardiovascular disease [1]. Progressive degeneration of microvascular beds is a major contributing factor to many complications of diabetes including retinopathy, nephropathy, and neuropathy. Macrovascular complications associated with diabetes include coronary heart disease, stroke, and peripheral vascular disease. The relationship between diabetes and vascular complications emphasizes the importance of understanding the mechanisms underlying this association. 

The increased risk of cardiovascular disease observed in diabetes is primarily due to a damaged or dysfunctional endothelium. In the diabetic state, numerous inflammatory, hormonal, and metabolic influences impinge upon the endothelium and alter its function. Elevated glucose and fatty acids lead to endothelial nitric oxide synthase (eNOS) uncoupling [2]. This in turn leads to reduced nitric oxide bioavailability and generation of reactive nitrogen and oxygen species as well as increased glycation of proteins and lipids [3–5]. Pathological levels of hormones and cytokines in the diabetic state alter endothelial signaling pathways promoting inflammation and atherogenesis and impairing vasoregulation and vascular hemostasis [6, 7]. Endothelial dysfunction precedes symptomatic vascular disease and is often detected before structural changes in the vascular wall [2]. A deeper understanding of the mechanisms underlying the endothelial response to diabetes should lead to new therapeutic interventions to inhibit these changes before they progress to vascular disease. It may also identify useful biomarkers of diabetic vasculopathy.

Endothelial isolation has been difficult, in part due to the lack of specific markers available for the endothelium. Column purification techniques often lead to contamination by other cell types. A freeze-fracture technique [8] and scraping of the endothelium off the vessel wall [9] usually do not achieve pure populations of endothelial cells and may cause damage leading to RNA degradation. These techniques may be acceptable if further selection is possible, such as altering cell culture conditions to eliminate contaminating cell types. However, *in vitro* responses of endothelial cells are highly dependent on the choice of growth medium and flow conditions [10]. *In vitro* studies are further complicated by the altered expression of endothelial cell markers and function commonly observed in both primary isolates and immortalized cultures [11]. Most importantly, monoculture of endothelial cells is a terrifically artificial environment lacking the hemodynamic stimuli, intercellular interactions, and hormonal influences of the intact, perfused, *in vivo *blood vessel. Therefore, assessment of the endothelial response to diabetes is optimally obtained with an *in vivo* murine model of diabetes accompanied by rapid isolation of pure populations of endothelial cells. 

We have previously described a high-fat diet-induced model of insulin resistance using Tie2-GFP mice wherein the endothelium can be reliably isolated by fluorescence-activated cell sorting based on Tie2-driven GFP expression and cell-surface staining for endothelial markers [12]. Here we have applied this high-fat model to two new strains of mice, ScN/Tie2-GFP and *ApoE*(−/−)/Tie2-GFP, created by interbreeding with Tie2-GFP mice. ScN/Tie2-GFP lack a functional toll-like receptor 4 (TLR4), which serves an important role in innate immunity and is the principal sensor of LPS [13]. TLR4 has also been implicated in promoting vascular insulin resistance by binding free fatty acids followed by subsequent activation of inflammatory pathways [14]. *ApoE*(−/−) mice lack the lipid carrier, apolipoprotein E, and consequently display hypercholesterolemia and increased susceptibility to the development of atherosclerosis. The crossing of TLR4- and APOE-deficient mice with Tie2-GFP mice allows the isolation of their endothelium in order to elucidate the molecular biology underlying these observed vascular phenotypes. Here we characterize the metabolic profiles of both strains after feeding a high-fat diet and compare their endocrine responses to that of Tie2-GFP mice. We also provide a detailed description of our endothelial isolation technique and other important considerations when studying the diabetic vasculature. 

## 2. Methods

### 2.1. Animals and Diet

Mice homozygous for the Tie2-green fluorescent protein (GFP) transgene (Tg(TIE2GFP)287Sato, stock number 003658 Jackson Laboratories (Bar Harbor, ME)) were bred for these experiments. Tie2-GFP mice were interbred with BL/6 *ApoE*(−/−) (strain name B6.129P2-*ApoE* ^tm1Unc^/J (stock number 002052)) and genetically selected to obtain *ApoE*(−/−)/Tie2-GFP mice on an approximately 1 : 1 mixed genetic background of C57BL/6 and FVB/N. Tie2-GFP mice were also crossed with BL/10 ScN (strain name C57BL/10ScNJ (stock number 003752)) and genetically selected to obtain ScN/Tie2-GFP mice on an approximately 1 : 1 mixed genetic background of C57BL/10 and FVB/N. For simplicity, ScN/Tie2-GFP and *ApoE*(−/−)/Tie2-GFP mice will be referred to as ScN/GFP and *ApoE*(−/−)/GFP, respectively. Beginning at 8 weeks of age, male mice were allowed to feed ad libitum on a high-fat diet (HFD) containing 60% fat calories (BioServ, Frenchtown, NJ, cat. number S3282) for a period up to 8 weeks. Littermates fed a normal chow diet containing 12% fat calories (LabDiet, St. Louis, MO, cat. number 5001) served as controls. All procedures were approved by the Institutional Animal Care and Use Committee of the University of Hawaii.

### 2.2. Measurement of Metabolic Characteristics

After 2, 4, 6, and 8 weeks on their respective diets, glucose levels were determined by glucometry of the tail blood following an overnight fast (OneTouch Ultra, Lifescan, Milpitas, CA, USA). A glucose tolerance test (GTT) was performed after 6 weeks on the diet regimen. Glucose (1 mg/g of body weight) was administered i.p. following an overnight fast. Glucometry of the tail blood was performed prior to glucose injection and every 20 minutes afterwards for 2 hrs. The area under the curve (AUC) was determined using the statistical software in Graphpad. After 8 weeks of HFD, insulin levels were measured in serum from overnight-fasted mice using a Mercodia Mouse Insulin ELISA kit (Uppsala, Sweden, cat. number 10-1149-01). Total cholesterol in the sera of overnight-fasted HFD-fed and chow-fed *ApoE*(−/−)/Tie2-GFP mice and both parental strains were measured by ELISA performed according to manufacturer's instructions (Cayman Chemical, Ann Arbor, MI, USA, cat. number 10007640).

### 2.3. Endothelial Isolation

Animals were sacrificed by CO_2_ asphyxiation and perfused with ice cold PBS. Aortae were dissected from the aortic root to the iliac bifurcation. Leg muscles consisting of the plantaris, gastrocnemius, and biceps femoris (which are readily dissected as a single group) were excised. Tissues were placed in cold PBS, and adherent fat was removed. The aortic and skeletal muscle tissues from 3 animals were each pooled, minced into 1 mm fragments, and dispersed in PBS with 100 mM CaCl_2_ and MgCl_2_ containing 5 mg/mL collagenase type 2 (Worthington Biochemical, Lakewood, NJ, cat. number LS004174), 2 mg/mL glucose, and 30 U/mL DNAse I (Worthington Biochemical, cat. number LS006331) under constant agitation (180 rpm at 37°C). Aortae were collagenolytically digested for 40 min, while the muscle was digested for 1 h, with mechanical dissociation obtained by triturating every 10 min. Enzyme activity was stopped by the addition of a 10% solution of FBS in PBS. Cells were pelleted by centrifugation (5 min, 1000 rpm, 4°C), aspirated, resuspended in PBS, and filtered through a 40 *μ*m cell strainer (BD Biosciences, San Jose, CA, USA, cat. number 352340). Red blood cells were lysed by resuspension in 1 mL RBC Lysis Solution (Applichem, St. Louis, MO, USA, cat. number A4617) overlaid on 4.5 mL FBS followed by centrifugation. In each experiment, pooled cells from 3 experimental and 3 control mice were collected for each tissue. 

Suspensions of collagenolytically separated cells were incubated with anti-mouse CD16/32 (1 : 500) for 5 min to prevent Fc binding, followed by phycoerythrin- (PE-) conjugated anti-mouse CD31 (1 : 200) for 25 minutes on ice (eBiosciences, San Diego, CA, cat. numbers 14-0161 and 12-0311). Cells were washed and resuspended in FACS buffer (PBS with 0.5 mM EDTA, 30 U/mL DNAse, 3% FBS, and 2 mg/mL glucose). Immediately before sorting, the cell suspension was passed through a CellTrics 30 *μ*m filter (Partec, Swedesboro, NJ, USA). Endothelial cells positive for both GFP and phycoerythrin staining were isolated with a FACSAria (Becton Dickinson, Franklin Lakes, NJ, USA). Cells were sorted directly into TRIzol (Invitrogen, Carlsbad, CA, USA) for transcriptional analyses or into medium (Endothelial Cell Growth Medium MV with supplement mix, PromoCell, Heidelberg, Germany, cat. number C-22020) for subsequent culturing or cytospin preparations.

### 2.4. Matrigel Tube Formation and Acetylated-Low Density Lipoprotein (ac-LDL) Uptake Assays

 GFP^+^ endothelial cells from the skeletal muscle were isolated by FACS and sorted into tissue culture medium for fluorescence microscopy and functional assays to confirm their endothelial origin. Aliquots containing 1000 cells were deposited onto lysine-coated slides by centrifugation at 450 rpm for 5 minutes with a Cytospin (Shandon). Cells were fixed in 10% formalin for 30 min and mounted in DAPI-containing medium. Cells were viewed under the fluorescent microscope and images were collected with an Axiophot system (Zeiss, Oberkochen, Germany). 

For tube formation assays, approximately 8,000 GFP^+^, sorted endothelial cells were plated per well in 48-well plates coated with Matrigel Basement Membrane Matrix (BD Biosciences, cat. number 354234) and allowed to grow in endothelial growth medium. After 5 days, tube formation was assessed. For ac-LDL uptake assays, the sorted endothelial cells were plated on Nunc Lab-Tek 8-well chamber slides (Thermo Scientific, Billerica, MA, USA, cat. number 154941) and incubated with 50 *μ*g/mL Dil-Ac-LDL (Biomedical Technologies Inc., Stoughton, MA, USA, cat. number BT-902) for 4 hours, washed, fixed in 2% paraformaldehyde, stained with DAPI, and imaged using the Axiophot system (Zeiss).

### 2.5. Flow Cytometry Analysis of Peripheral Blood Mononuclear Cells (PBMCs)

Approximately 200 *μ*L of blood was collected from the retro-orbital vein of chow- and HFD-fed mice after 8 weeks of feeding into EDTA-coated tubes. RBCs were lysed using a 1 : 10 dilution of BD Pharm Lyse (cat. number 555899). Leukocytes were stained for 20 min at 4°C with 50 *μ*L of antibody cocktail containing 0.5 *μ*L each of PE-conjugated anti-mouse CD115 (eBiosciences, cat. number 12-1152), peridinin chlorophyll protein-cyanine 5.5- (PerCP-Cy5.5-) conjugated anti-CD11b (BD Biosciences, cat. number 550993), allophycocyanin-cyanine 7 (APC-Cy7) anti-CD45 (BD Biosciences, cat. number 557659), and PE-Cy7 anti-CD31 (eBiosciences, cat. number 25-0311) diluted in a 1 : 1 solution of Hanks: FACS staining buffer (1.7% BSA, 0.02% mouse serum, 0.02% rabbit serum, and 0.02% human serum). After washing, samples were fixed for 20 min at RT in a 1 : 1 solution of Hanks: fixation buffer (Biolegend, San Diego, CA, USA, cat. number 420801). Flow cytometry was performed on a FACSAria, and CD45^+^ leukocytes, CD45^+^/CD11b^+^ myeloid cells, and CD45^+^/CD11b^+^/CD115^+^ monocytes were analyzed for GFP signal using FlowJo software (Tree Star, Inc., Ashland, OR, USA).

### 2.6. Flow Cytometry Analysis of Endothelial Populations Stained for Monocyte Markers

Collagenolytically separated cell suspensions derived from aortic and muscle tissues as previously described were stained for 25 min at 4°C with Alexa Fluor^®^ 647 anti-mouse CD11b (eBiosciences, cat. number 51-0112), and flow cytometry was performed on a FACSAria. The sorting gates were established by initially gating for mononuclear cells based on forward scatter. This was followed by gating for GFP fluorescence, which was considered to be any signal in the FITC channel above that of an unstained, non-GFP sample. After applying the sorting gate for the GFP^+^ endothelial cells, the number of GFP^+^/CD11b^+^ events within that gate was analyzed using FlowJO software. 

### 2.7. Microarray Analysis

Microarray analyses were performed to determine the transcriptional responses in aortic and skeletal muscle endothelium from *ApoE*(−/−)/GFP mice exposed to high-fat diet for 4 weeks compared to *ApoE*(−/−)/GFP mice fed a chow diet. 10,000 endothelial cells positive for both GFP and phycoerythrin-CD31 staining were isolated with a FACSAria directly into TRIzol. RNA was purified with RNeasy columns (Qiagen, Valencia, CA, USA) to yield <80 ng, which was then amplified using an Ambion Amino Allyl MessageAmp kit (Life Technologies, Carlsbad, CA, USA, cat. number AM1753) according to the manufacturer's protocol to produce approximately 100 *μ*g of amino-allyl modified cRNA. This was labeled with Cy3 and Cy5 CyDye Post-Labeling Reactive Dye Pack (GE Healthcare, Waukesha, WI, USA) according to the manufacturer's instructions. Following purification, 200 pmoles of Cy3 and Cy5 dye-labeled cRNA, as measured by NanoDrop 2000c spectrophotometer (Thermo Scientific), were combined and fragmented with Ambion 10X fragmentation reagents at 70°C for 15 min. Yeast tRNA (4 *μ*g), polyA RNA (4 *μ*g), mouse cot-1 DNA (1 *μ*g), and Slidehyb III hybridization buffer (Ambion) were added for a total sample volume of 35 *μ*L. Triplicate experiments were performed for both tissues. For each of the biological replicate experiments, two array hybridizations were performed, each of which included a dye reversal. Samples were hybridized overnight to glass slides spotted with the Operon Murine V4 oligo set produced by the Duke University Microarray Core. Arrays were scanned using a Genepix 4000B system (Molecular Devices, Union City, CA, USA) and analyzed with Genepix and Acuity software.

Statistical analysis of microarray results was performed using the functions available within Acuity. Results were normalized using the ratio of medians method and filtered to exclude any exhibiting the following characteristics: a percentage of saturated pixels >3, a signal/noise ratio <3, a (regression ratio 635/532)^2^ <0.6, or a Genepix flag. Transcripts exhibiting upregulation (a log_2_(fold change) >0.75) or downregulation (log_2_(fold change) <−0.75) were tabulated for each tissue. The one sample *t*-test function within Acuity was then applied to the results to calculate *P* values for each transcript.

## 3. Results and Discussion

### 3.1. Murine Model of Type II Diabetes

In order to provide a more authentic model of the pathophysiological response observed in the human population, we have chosen to use a nutritional model of type II diabetes induced by feeding a high-fat diet consisting of 60% fat calories. The compositions of the high-fat and chow diets are compared in Table 1. The high-fat diet contains nearly 60% of its calories from fat, while the normal chow diet derives 60% of its calories from carbohydrates and only 12% of its calories from fat (Table 1). The total caloric intake per gram is much greater in the HFD compared to the chow diet (Table 1). Therefore, mice consuming equal quantities of the diet receive more calories from the HFD than the chow diet, and this is further exacerbated by the hyperphagia induced by the HFD [15]. Studies have also shown that calories derived from fat are more diabetogenic than the same caloric intake from carbohydrates [16]. In humans, dietary fat intake has been linked to obesity, decreased insulin sensitivity, and progression to type II diabetes [17–19]. High-fat diets also raise LDL cholesterol and increase LDL particle size compared to low-fat diets in human subjects [20]. 

This diabetic model allows us to evaluate the endothelial response to the diabetic milieu as a whole without selecting for particular biochemical attributes of diabetes. However, this also means that individual responses of the endothelium cannot be definitively ascribed to one aspect of diabetes or another, such as those changes due to insulin resistance versus hyperglycemia versus weight gain, and so forth. However, we can try to assign causation based on our current understanding of endothelial cell biology and the effects of diabetes in other cell types. The use of knockout models, however, does allow us to determine differential vascular responses due to a specific gene deletion. Analysis of ScN/GFP and *ApoE*(−/−)/GFP endothelial responses to this high-fat diet provides insight into the effect of TLR4 and APOE deficiency. 

### 3.2. Metabolic Characteristics of ScN/GFP Mice on a High-Fat Diet Regimen

**Figure 1:**
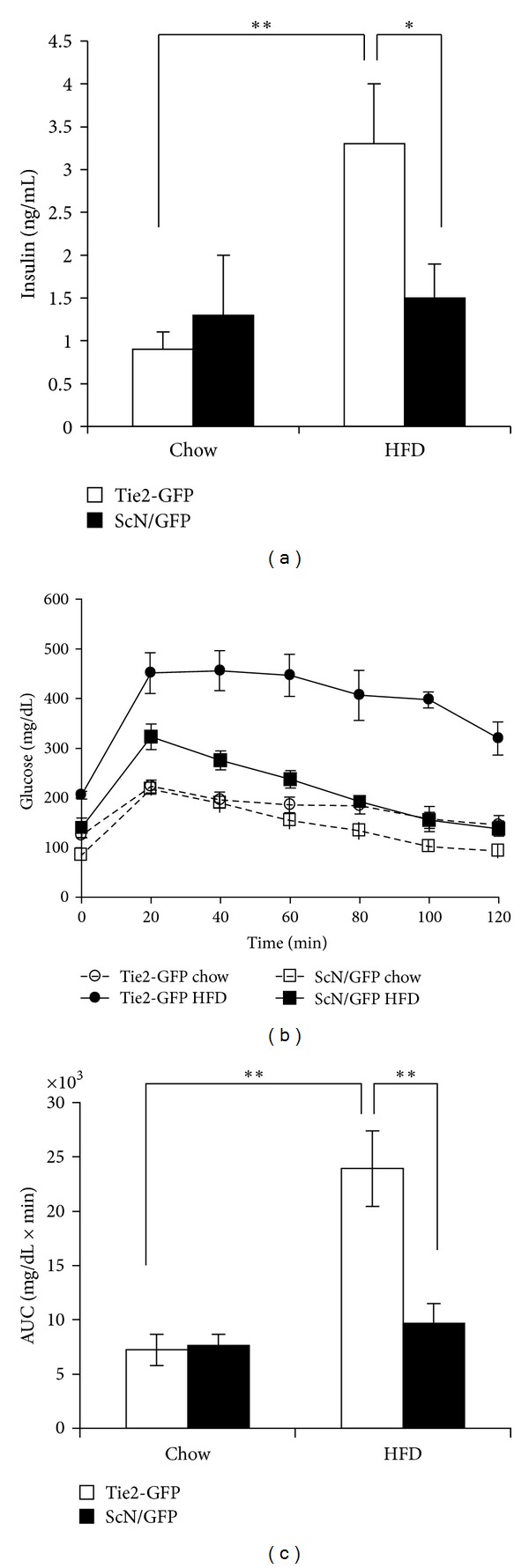
(a) Fasting insulin levels of ScN/GFP and Tie2-GFP mice after 8 weeks of HFD or chow diet (*n* = 3–9). (b) Following 6 weeks of HFD, glucose tolerance was determined by performing glucometry over a 2 h period after an intraperitoneal injection of glucose (1 mg/g) following an overnight fast (*n* = 5-6). (c) Area under the curve of the glucose tolerance test. The data are presented as means ± SE. *P < 0.05 and **P ≤ 0.01.

As we have previously described, Tie2-GFP mice have accelerated weight gain and hyperglycemia by 2 weeks, marked hyperinsulinemia and impaired glucose tolerance by 6 weeks, and aortic vascular insulin resistance observed after 5 weeks of HF feeding [12]. ScN/GFP mice on chow diet have lower fasting glucose levels compared to Tie2-GFP mice (Table 2). Over the time course of the feeding, the weights and fasting glucose levels of ScN/GFP mice increase compared to chow-fed controls (Table 2). Although elevated compared to chow-fed controls, the fasting glucose levels of ScN/GFP mice remain significantly lower than that of Tie2-GFP mice even after 8 weeks of HFD (Table 2). Insulin levels of ScN/GFP mice are also much lower compared to Tie2-GFP mice and are not different from chow-fed controls (Figure 1(a)). Glucose tolerance testing of ScN/GFP mice after 6 weeks of HFD reveals a normal response to glucose challenge compared to Tie2-GFP HFD mice where glucose tolerance is impaired (Figures 1(b) and 1(c)).

Obese and type II diabetic patients display increased expression of TLR4 in their skeletal muscle, implicating its role in diabetic pathophysiology [21]. Following 8 weeks of high-fat diet, whole aorta lysates from C57BL/6 mice have been shown to display decreased phosphorylation of protein kinase B (AKT) and endothelial nitric oxide synthase (eNOS) after insulin stimulation, suggesting a state of vascular insulin resistance and impaired vasoregulation, which were not observed in TLR4(−/−) mice [14]. Our studies also suggest that TLR4 serves a role in the development of insulin resistance as its deficiency in ScN/GFP mice lowers glucose and insulin levels and improves glucose tolerance. The ability to isolate the endothelium from these mice allows molecular studies to elucidate the mechanisms underlying the role of TLR4 in vascular insulin resistance and endothelial dysfunction.

### 3.3. Metabolic Characteristics of *ApoE*(−/−)/GFP Mice on a High-Fat Diet Regimen

**Figure 2:**
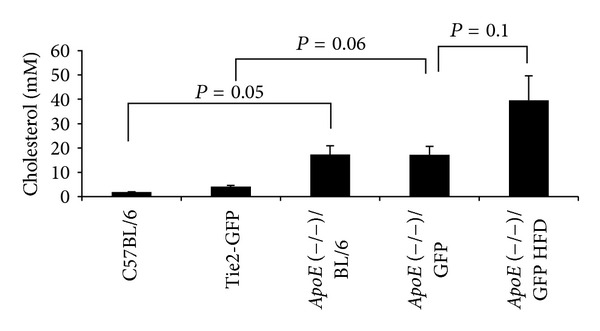
Total serum cholesterol levels of *ApoE*(−/−)/GFP mice on chow or HFD compared to that of the parental strains. Data is shown as mean ± SE (*n* = 3).

*ApoE*(−/−)/GFP mice also display increased weight gain after HFD feeding (Table 2). Unlike ScN/GFP mice, the fasting glucose levels of *ApoE*(−/−)/GFP mice are elevated compared to Tie2-GFP mice (Table 2). Over the time course of the feeding, the fasting glucose levels of *ApoE*(−/−)/GFP mice on HFD increase slightly compared to chow-fed controls. *ApoE*(−/−)/BL/6 mice are hypercholesterolemic due to defective lipoprotein metabolism. We therefore assessed the total cholesterol levels in the sera of *ApoE*(−/−)/GFP mice and found that they exhibit similar cholesterol levels to *ApoE*(−/−) mice on the C57BL/6 background. After 5 months of HF feeding, these levels doubled (Figure 2).

### 3.4. Choice of Endothelial Population

To assess macrovascular responses, we isolate the endothelium of the largest artery, the aorta. To assess microvascular responses, we isolate the leg muscle. The vasculature of the skeletal muscle is mostly composed of small arterioles and venules of capillary beds with some larger arteries and veins feeding into them. Therefore, the endothelial cells isolated from the muscle are a heterogeneous mixture of arterial, venular, and lymphatic endothelial populations mostly derived from microvessels. To date, there are no acceptable antibodies to specifically isolate these endothelial subtypes although the differential expression of a few genes has been identified [22].

The heterogeneity of endothelial populations among the different vascular beds suggests that the responses of any one vascular bed may be not be reflective of those of the entire circulatory system. We have previously performed gene expression analyses on the endothelium derived from two vascular beds of Tie2-GFP mice and have identified both the specific and common responses of the large vessel endothelium and the microvasculature to the diabetic state [12]. The transcriptional responses of the macrovascular and microvascular endothelial cells to diabetes differ in both the degree and onset of dysregulation. While some transcripts are commonly regulated among the 2 vascular beds, others are specific to one vascular bed. Therefore, the choice of endothelial population is an important consideration when studying endothelial responses and vascular complications of diabetes. 

### 3.5. Endothelial Isolation by Fluorescence-Activated Cell Sorting

In Tie2-GFP mice, GFP expression is driven by the endothelial promoter for the angiopoietin receptor, Tie2, which is important in angiogenesis and vasculogenesis [23]. Therefore, all endothelial cells of Tie2-GFP mice fluoresce at a peak wavelength of 509 nm when excited at a wavelength of 488 nm. This allows for endothelial cell isolation by fluorescence-activated cell sorting (FACS). 

**Figure 3:**
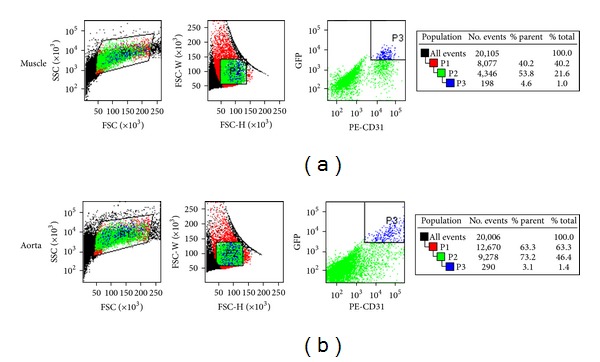
FACS isolation of endothelium. FACS profiles of collagenolytic digests of leg muscle (a) or aorta (b) from ScN/GFP mice. Cell suspensions from ScN/GFP tissues were labeled with phycoerythrin-*α*CD31. Cells are first gated by size and granularity based on forward and side scatter pulse area (FSC/SSC, gate P1) followed by further selection for mononuclear cells based on forward scatter pulse width and height (FSC-W/FSC-H, gate P2). Cells expressing CD31 and GFP are then gated by phycoerythrin and GFP fluorescent signals as determined based on unstained samples. GFP^+^/CD31^+^ endothelial cells shown in gate P3 are sorted on a FACSAria directly into TRIzol or media for subsequent analysis.

Fat in tissue digests is highly autofluorescent and is particularly abundant in diabetic models. The identification of GFP^+^ endothelial cells is thus enhanced by double staining for CD31/Platelet endothelial cell adhesion molecule 1 (PECAM-1), which does not label fat cells. CD31 is highly expressed by endothelial cells and has long been used as a marker for endothelial cell isolation [24]. However, it is also expressed by leukocytes and therefore, may not be used alone. As shown in Figure 3, all GFP^+^ cells also exhibit phycoerythrin-CD31 staining, whereas an additional population exhibits CD31 staining without GFP fluorescence. 

GFP^+^/CD31^+^ endothelial cell populations represent approximately 1-2% of the total population of cells derived from muscle and aortic tissue (Figure 3). Approximately 3,500 endothelial cells may be isolated from one mouse aorta and up to 30,000 cells from the skeletal muscle prep described in this study. We routinely collect at least 10,000 GFP^+^/CD31^+^ cells from each pooled tissue sample for transcriptional analysis. 

### 3.6. Confirmation of Endothelial Identity and Assessment of Monocyte Contamination

**Figure 4:**
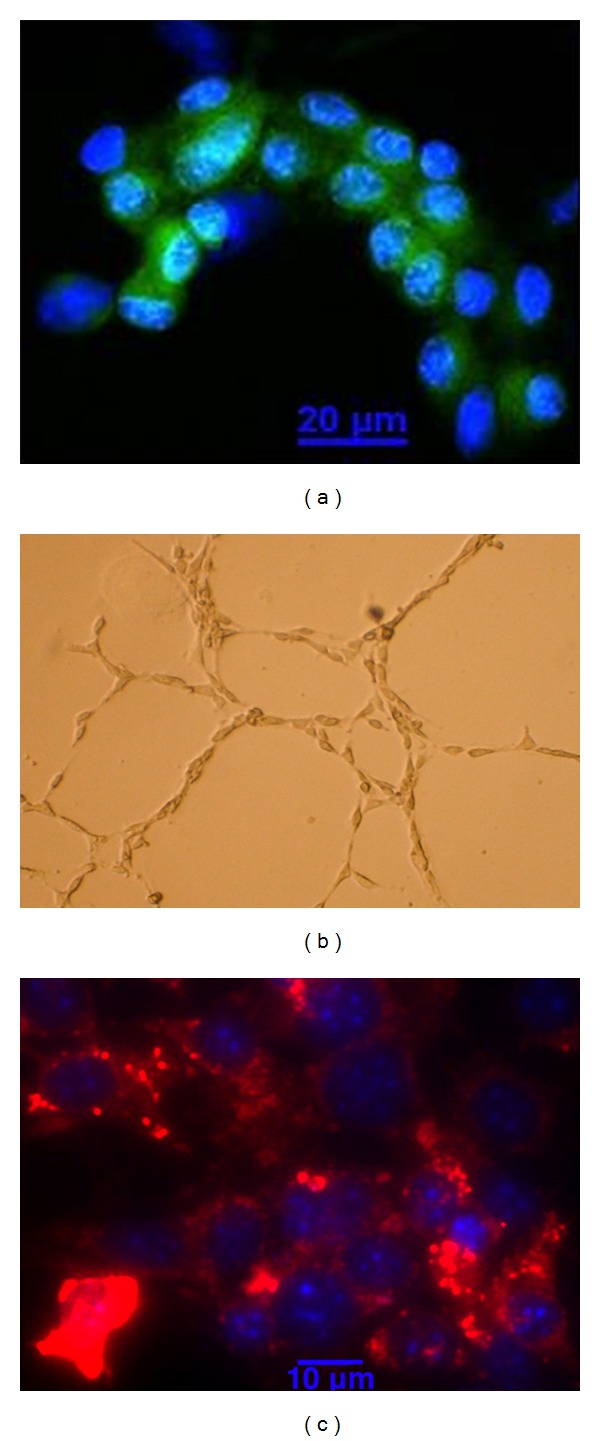
Confirmation of endothelial identity. GFP^+^ cells sorted by FACS were collected directly into media. (a) Cells were spun onto slides and fixed for viewing by fluorescence microscopy to confirm the presence of only GFP^+^ cells. (b) Sorted cells form tubular structures after 5 days when plated on matrigel. (c) Sorted cells cultured for 5 days take up Dil-Ac-LDL after a 4 h incubation.

The endothelial identity of the sorted cells is confirmed by fluorescence microscopy. After FACS, we see only GFP^+^ endothelial cells and no other contaminating, non-GFP cells (Figure 4(a)). Endothelial identity is further confirmed by functional assays. GFP^+^ cells sorted from the skeletal muscle retain the unique endothelial ability to form tubes when plated on Matrigel (Figure 4(b)). In addition, the sorted cells metabolize acetylated-LDL as demonstrated by Dil fluorescence, a property that is specific to endothelial cells and macrophages (Figure 4(c)). 

**Figure 5:**
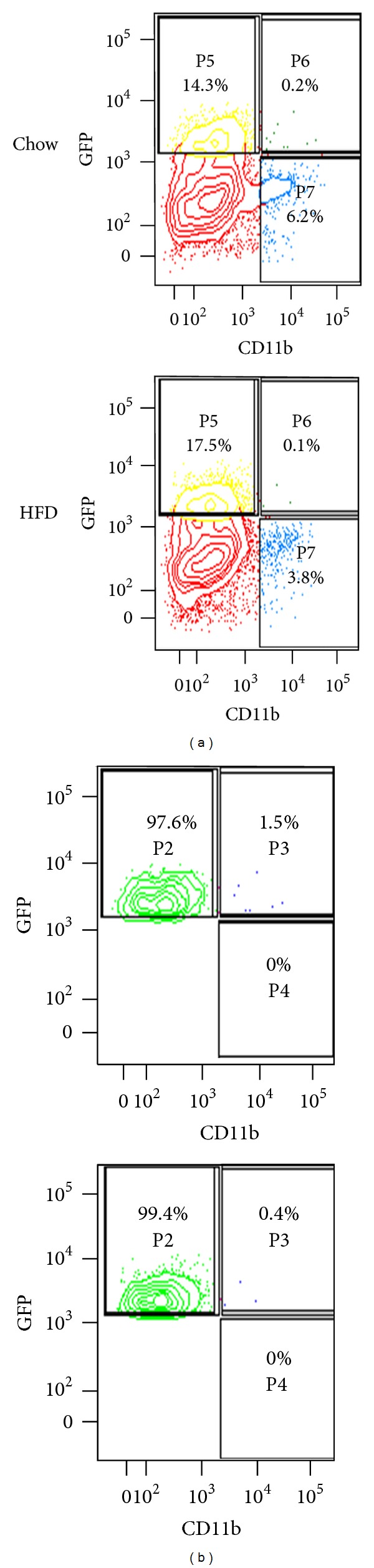
Flow cytometry analysis of monocyte marker expression in the sorted endothelial population. Cells from the skeletal muscle of ScN/GFP mice fed chow or HFD for 8 weeks were stained for the monocyte marker CD11b. (a) Percentage of all mononuclear cells that are GFP^+^/CD11b^−^, GFP^+^/CD11b^+^, or GFP^−^/CD11b^+^ are shown in gates P5–P7, respectively. (b) Percentage of the sorting population that are GFP^+^/CD11b^−^ or GFP^+^/CD11b^+^ are indicated in gates P2 and P3. The sorting gate is established based on GFP^+^ fluorescent signal above non-GFP sample. There are no GFP^−^/CD11b^+^ cells within the sorting gate as shown in gate P4. Only GFP^+^ cells with no fluorescent signal for CD11b are present in unstained samples from ScN/GFP mice (not shown).

Tie2-expressing myeloid cells have been reported to account for 2–7% of human blood mononuclear cells [25], which also express CD11b. We analyzed the number of CD11b^+^ events within our sorting gates by flow cytometry of muscle tissue suspensions from 3 independent experiments following 8 weeks of diet. In Tie2-GFP mice, FACS revealed that only 0.8 ± 0.4% of GFP^+^ cells derived from HFD animals also display the monocyte marker CD11b with similar low percentages observed in control animals (0.3 ± 0.5%). Similarly, analysis of GFP^+^ cells derived from ScN/GFP mice reveals few GFP^+^ cells staining for CD11b in pooled samples from either control or HFD mice (Figure 5).

In diabetes, there exists a state of inflammation characterized by an increase in activated monocytes in the peripheral blood of diabetic patients [26]. Analysis of the CD11b^+^/CD115^+^ inflammatory monocyte population in peripheral blood reveals a nonsignificant increase from 9 ± 2% of CD45^+^ leukocytes in chow-fed mice to 11 ± 3% in Tie2-GFP mice fed HFD for 8 weeks (Table 3). We therefore investigated whether the number of monocytes appearing to have a positive signal for GFP was altered by HFD. As shown in Table 3, chow and HFD mice display similar, low levels of GFP^+^ cells in the peripheral blood. Importantly, this “GFP^+^” signal is much lower than that obtained from the endothelial population and would therefore be excluded by gating. 

### 3.7. Sorted Endothelial Cells Are Suited for Gene Expression Analysis

The endothelial cell sorting technique described here is useful for transcriptional studies of endothelial dysfunction where pure populations of endothelial cells are necessary. We have previously analyzed the endothelial response to diabetes in the aortic and muscle endothelium of Tie2-GFP mice. Here we have performed similar studies on APOE-deficient mice in order to determine the effect of preexisting hypercholesterolemia on the endothelial response to diabetes. While Tie2-GFP mice have 209 transcripts dysregulated >2-fold in the aortic endothelium by 4 weeks of HFD compared to chow-fed controls, *ApoE*(−/−)/GFP mice on HFD have only 32 transcripts dysregulated to this degree compared to chow-fed *ApoE*(−/−)/GFP controls. Thus, the endothelial response to the diabetic milieu is blunted in *ApoE*(−/−)/GFP mice, most likely due to preexisting endothelial activation in the hypercholesterolemic state. However, some transcripts, such as vascular cell adhesion molecule 1 (VCAM-1) and insulin-like growth factor II (Igf-II), are differentially regulated in *ApoE*(−/−)/GFP mice after HFD compared to chow-fed controls. Transcripts dysregulated in the aortic endothelium are shown in Table 4(a) and those dysregulated in the skeletal muscle are presented in Table 4(b). Similar transcriptional analyses may be performed on ScN/GFP mice to elucidate the role of TLR4 in vascular insulin resistance and endothelial dysfunction upon high-fat feeding.

### 3.8. Potential Limitations

The use of the *Tie2-GFP* transgene, which is expressed at moderately high levels, may potentially affect endothelial function, either by nonspecific effects of a novel protein or, perhaps, by specific squelching effects of the *Tie2* promoter on expression of the endogenous *Tie2* gene. We have not seen any gross effects upon the phenotyping described in this study; however, we cannot exclude subtle effects. It is thus ultimately useful to confirm results from the Tie2-GFP studies in nontransgenic models. For example, transcriptional regulation identified in FACS-sorted cells can be confirmed by immunohistology or *in situ* hybridization on nontransgenic vascular samples.

## 4. Conclusions 

We have demonstrated the feasibility of isolating vascular endothelial cells from murine models of diabetes in order to study responses of the diabetic vasculature. We have shown that the endothelium may be isolated from these mice by FACS of GFP^+^/CD31^+^ cells. Flow cytometry analyses, fluorescence microscopy, and functional studies have shown the sorted cells to be pure populations of endothelial cells with minimal contamination by other cell types. This sorting technique may be applied to isolate the endothelium of any strain interbred with Tie2-GFP mice.

 In this study, we describe the metabolic responses and endothelial isolation of ScN/GFP and *ApoE*(−/−)/GFP mice. ScN/GFP mice display decreased susceptibility to the development of insulin resistance by HFD as demonstrated by lower fasting glucose and insulin levels and improved glucose tolerance compared to Tie2-GFP mice. TLR4 has been shown to play a role in vascular insulin resistance due to its ability to bind free fatty acids and activate inflammatory pathways [14]. Therefore, this model of high-fat feeding and endothelial isolation may be useful to explore the metabolic and vascular changes due to TLR4 deficiency. *ApoE*(−/−)/GFP mice display elevated glucose and cholesterol levels compared to Tie2-GFP mice. Transcriptional analyses of the endothelium isolated from the aorta and skeletal muscle of these mice demonstrate reduced endothelial response to HFD, perhaps resulting from endothelial dysfunction in the preexisting hypercholesterolemic state in chow-fed *ApoE*(−/−)/GFP mice. Similar studies to evaluate the effect of gene loss on the diabetic endothelium and vascular responses may be performed by utilizing the techniques described in this study.

## Figures and Tables

**Table 1:** Composition of high-fat diet (Bioserv S3282) and control diet (LabDiet 5001).

Nutritional component	Composition by weight (%)	Calories (kcal/g)	% Calories
Fat	36	3.2	59
Carbohydrate	35.7	1.4	26
Protein	20.5	0.8	15

Total		5.5	

Fat	4.5	0.40	12
Carbohydrate	49.9	2.0	60
Protein	23.4	0.9	28

Total		3.3	

**Table 2:** Weights and fasting glucose levels of Tie2-GFP, ScN/GFP, and *ApoE*(−/−)/GFP mice receiving a high-fat diet versus chow diet.

Weeks	Tie2-GFP		ScN/GFP		*ApoE*(−/−)/GFP	
on diet	Chow	HFD	Chow	HFD	Chow	HFD
Weight (g)						
2	24.1 ± 0.8 (12)	30.0 ± 1.6 (14)**	20.5 ± 0.4 (4)^†^	24.9 ± 0.9 (5)^∗∗*∧*^	23.1 ± 2.0 (3)	31.5 ± 0.8 (7)*
4	26.8 ± 0.8 (18)	31.1 ± 1.3 (19)**	22.7 ± 0.8 (7)^‡^	26.7 ± 1.1 (7)^∗∗*∧*^	26.6 ± 0.9 (13)	33.0 ± 0.9 (16)***
6	26.3 ± 0.8 (14)	33.8 ± 1.2 (15)***	25.5 ± 0.8 (12)	28.4 ± 0.6 (12)^∗∗‡^	27.7 ± 1.2 (11)	34.7 ± 1.2 (12)***
8	28.0 ± 0.9 (14)	36.5 ± 1.0 (15)***	24.0 ± 0.3 (3)^‡^	33.0 ± 1.5 (3)*	25.2 ± 1.7 (8)	33.7 ± 2.3 (7)*
Glucose (mg/dL)						
2	148 ± 9 (12)	190 ± 10 (14)**	85 ± 7 (4)^‡^	117 ± 11 (5)^∗‡^	152 ± 17 (3)	168 ± 19 (7)
4	116 ± 8 (16)	163 ± 9 (17)***	82 ± 14 (3)	131 ± 9 (7)^∗†^	144 ± 8 (6)^*∧*^	184 ± 13 (10)*
6	139 ± 9 (18)	210 ± 10 (18)***	85 ± 5 (10)^‡^	118 ± 9 (10)^∗∗‡^	137 ± 18 (7)	157 ± 10 (7)^†^
8	111 ± 4 (11)	182 ± 4 (9)***	76 ± 15 (5)	137 ± 5 (5)^∗∗‡^	160 ± 2 (3)^‡^	212 ± 3 (3)^∗∗∗‡^

Data are means ± SE. Weight measurements and glucometry of the tail blood were performed after 2, 4, 6, and 8 weeks of feeding. *N*'s are indicated in parentheses.*P < 0.05, **P ≤ 0.01, and ***P < 0.001 versus chow-fed controls; ^*∧*^ *P* < 0.05, ^†^ *P* ≤ 0.01, and ^‡^ *P* < 0.001 versus Tie2-GFP.

**Table 3:** Flow cytometry analysis of Tie2-GFP expression in peripheral blood mononuclear cells (PBMCs) derived from Tie2-GFP mice fed a high-fat or chow diet for 8 weeks.

	CD11b^+^ (% of CD45^+^)	CD11b^+^/CD115^+^ (% of CD45^+^)	GFP^+^ (% of CD45^+^/CD11b^+^)	GFP^+^ (% of CD45^+^/CD11b^+^/CD115^+^)
Chow	30 ± 4	9 ± 2	0.6 ± 0.1	0.8 ± 0.3
HFD	33 ± 6	11 ± 3	0.6 ± 0.2	0.8 ± 0.5

PBMCs were isolated and stained for CD45, CD11b, and CD115. The population positive for the common leukocyte antigen, CD45, was analyzed for CD11b^+^ myeloid cells and CD11b^+^/CD115^+^ monocytes. The percentage of these populations appearing to have a positive signal for GFP is shown. Data is presented as mean ± SD (*n* = 3).

**Table:** (a)

RefSeq	Description	Gene symbol	Log_2_ fold change	*P* value
NM_008134	Glycosylation-dependent cell adhesion molecule 1	Glycam1	2.539	0.01
NM_010382	Histocompatibility 2, class II antigen E beta	H2-Eb1	2.22	0.01
NM_011498	Basic helix-loop-helix domain containing, class B2	Bhlhb2	1.388	0.05
NM_011693	Vascular cell adhesion molecule 1	Vcam1	1.377	0.03
NM_019414	Selenium-binding protein 2	Selenbp2	1.228	0.04
NM_007929	Epithelial membrane protein 2	Emp2	1.211	0.02
NM_013904	Hairy/enhancer-of-split-related with YRPW motif 2	Hey2	1.188	0.05
NM_023117	Cell division cycle 25 homolog B (S. cerevisiae)	Cdc25b	1.164	0.04
NM_145144	RIKEN cDNA 2810003C17 gene	2810003C17Rik	1.112	0.02
XM_128002	Collagen triple helix repeat containing 1	Cthrc1	1.095	0.01
NM_007554	Bone morphogenetic protein 4	Bmp4	1.082	0.01
NM_175397	RIKEN cDNA 5830484A20 gene	5830484A20Rik	1.042	0.02
XM_484254	Extracellular matrix protein 2	Ecm2	1.032	0.03
NM_008357	Interleukin 15, transcript variant 1, mRNA	Il15	0.993	0.05
NM_022004	FXYD domain-containing ion transport regulator 6	Fxyd6	0.976	0.03
XM_133801	Golgi associated, gamma adapt-in ear containing, ARF-binding protein 2	Gga2	0.933	0.03
NM_138587	Family with sequence similarity 3, member C	fam3c	0.924	0.04
NM_025809	C-type lectin domain family 14, member a	Clec14a	0.911	0.02
NM_013750	Pleckstrin homology-like domain, family A, member 3	Phlda3	0.899	0.03
NM_008330	Interferon gamma inducible protein 47	Ifi47	0.892	0.04
XM_140320	Coiled-coil domain containing 112	Ccdc112	0.879	0.03
NM_133663	Integrin beta 4	Itgb4	0.879	0.02
NM_025443	Partner of NOB1 homolog (*S. cerevisiae*)	Pno1	0.875	0.03
NM_007722	Chemokine (C-X-C motif) receptor 7	Cxcr7	0.875	0.01
XM_485110	RIKEN cDNA 2210418O10 gene	2210418O10Rik	0.87	0.02
NM_009463	Uncoupling protein 1 (mitochondrial, proton carrier)	Ucp1	0.857	0.04
NM_177618	cDNA sequence BC030477	BC030477	0.856	0.02
NM_183259	RIKEN cDNA 2210020M01 gene	2210020M01Rik	0.83	0.02
NM_010514	Insulin-like growth factor 2	Igf2	0.819	0.04
NM_010424	Hemochromatosis	Hfe	0.815	0.03
NM_177325	TSR1, 20S rRNA accumulation, homolog (yeast)	Tsr1	0.814	0.02
NM_001004062	CREB-regulated transcription coactivator 1	Crtc1	0.802	0.01
NM_133964	Deoxyhypusine hydroxylase/monooxygenase	Dohh	0.797	0.00
NM_212445	KDEL (Lys-Asp-Glu-Leu) containing 2	Kdelc2	0.795	0.03
NM_027334	UbiE-YGHL1 fusion protein	Ubie	0.768	0.02
NM_008610	Matrix metallopeptidase 2	Mmp2	0.765	0.03
NM_009024	Retinoic acid receptor, alpha	Rara	0.759	0.04
NM_008659	Myosin IC	Myo1c	−0.818	0.00
NM_001002239	Ribosomal protein L17, mRNA	Rpl17	−0.85	0.02
NM_146011	Rho GTPase activating protein 9	Arhgap9	−0.872	0.01
NM_020261	Pregnancy-specific glycoprotein 23	Psg23	−0.874	0.01
NM_016892	Copper chaperone for superoxide dismutase	Ccs	−0.905	0.02
NM_021469	Dysferlin	Dysf	−0.943	0.04
NM_027015	Ribosomal protein S27	Rps27	−1.174	0.02
NM_010826	MRV integration site 1	Mrvi1	−1.218	0.01
NM_007913	Early growth response 1	Egr1	−1.423	0.01
NM_017398	Diaphanous homolog 2 (*Drosophila*)	Diap2	−1.485	0.04

**Table:** (b)

RefSeq	Description	Gene symbol	Log_2_ fold change	*P* value
NM_025557	Purkinje cell protein 4-like 1	Pcp4l1	1.988	0.01
NM_008250	H2.0-like homeo box 1 (*Drosophila*)	Hlx1	1.544	0.05
NM_013549	Histone cluster 2, H3c2	Hist2h3c2	1.536	0.01
NM_011620	Troponin T3, skeletal, fast	Tnnt3	1.426	0.04
NM_029688	Sulfiredoxin 1 homolog (*S. cerevisiae*)	Srxn1	1.335	0.03
NM_026095	Small nuclear ribonucleoprotein D3	Snrpd3	1.277	0.04
NM_010382	Histocompatibility 2, class II antigen E beta	H2-Eb1	1.264	0.02
NM_008162	Glutathione peroxidase 4	Gpx4	1.221	0.02
XM_488375	Similar to barrier to autointegration factor 1 (LOC385407)	—	1.215	0.02
NM_007507	ATP synthase, H+ transporting, mitochondrial F1F0 complex, subunit e	Atp5k	1.199	0.01
NM_011313	S100 calcium-binding protein A6 (calcyclin)	S100a6	1.197	0.02
XM_128110	Polymerase (RNA) II (DNA directed) polypeptide F	Polr2f	1.195	0.00
NM_207648	Histocompatibility 2, Q region locus 6	H2-Q6	1.176	0.01
NM_010361	Glutathione S-transferase, theta 2	Gstt2	1.166	0.01
NM_181316	RIKEN cDNA E130103I17 gene	E130103I17Rik	1.136	0.03
NM_011119	Proliferation-associated 2G4	Pa2g4	1.119	0.03
NM_153152	RIKEN cDNA 2410015M20 gene	2410015M20Rik	1.086	0.01
NM_053078	DNA segment, human D4S114 (D0H4S114), transcript variant 1	—	1.065	0.04
NM_013706	CD52 antigen	Cd52	1.047	0.02
NM_026616	Ribonuclease H2, subunit C	Rnaseh2c	1.044	0.01
NM_011289	Ribosomal protein L27	Rpl27	1.02	0.01
XM_109683	RIKEN cDNA 1810027O10 gene	1810027O10Rik	1.006	0.02
NM_018832	PDZ domain containing, X chromosome	Pdzx	1.003	0.02
NM_024171	Sec61 beta subunit	Sec61b	0.95	0.02
NM_009081	Ribosomal protein L28	Rpl28	0.95	0.04
NM_008210	H3 histone, family 3A	H3f3a	0.942	0.02
XM_193800	RIKEN cDNA 1500032L24 gene	1500032L24Rik	0.927	0.01
XM_489638	Ribosomal protein L26	Rpl26	0.91	0.04
NM_021407	Triggering receptor expressed on myeloid cells 3	Trem3	0.872	0.02
NM_009811	Caspase 6	Casp6	0.858	0.04
NM_011185	Proteasome (prosome, macropain) subunit, beta type 1	Psmb1	0.855	0.01
NM_008303	Heat shock protein 1 (chaperonin 10), related sequence 1	Hspe1-rs1	0.84	0.00
NM_019682	Dynein light chain LC8-type 1	Dynll1	0.836	0.04
XM_138368	Similar to Rpl7a protein (LOC217924)	—	0.816	0.02
NM_010174	Fatty-acid-binding protein 3, muscle and heart	Fabp3	0.813	0.03
NM_175399	Exosome component 4	Exosc4	0.81	0.04
NM_013498	Camp-responsive element modulator	Crem	0.802	0.00
NM_010394	Histocompatibility 2, Q region locus 7	H2-Q7	0.793	0.03
NM_011129	Septin 4	39329	0.785	0.04
NM_025396	6-Phosphogluconolactonase	Pgls	0.783	0.05
NM_212470	RIKEN cDNA 0610007C21 gene	0610007C21Rik	0.779	0.02
XM_133877	RIKEN cDNA 4933402N03 gene	4933402N03Rik	0.774	0.02
NM_011343	SEC61, gamma subunit	Sec61g	0.77	0.02
NM_025946	RIKEN cDNA 2010100O12 gene	2010100O12Rik	0.767	0.05
NM_025338	Aurora kinase A interacting protein 1	Aurkaip1	0.761	0.01
NM_009077	Ribosomal protein L18	Rpl18	0.753	0.05
